# Supplementary material for: The Response Surface Optimization of Supercritical CO2 Modified with Ethanol Extraction of p-Anisic Acid from Acacia mearnsii Flowers and Mathematical Modeling of the Mass Transfer
Source: Molecules. 2022 Jan 31;27(3):970. doi: 10.3390/molecules27030970 (PMC8840752; doi:10.3390/molecules27030970)
Supplement: Supplementary file 1 [file molecules-27-00970-s001.zip › molecules-1532240-supplementary.pdf]

Support information

Figures S1-S7 below exhibit the chromatograms of the 7 crude extracts obtained with factorial design. Extraction conditions for each extract are shown in parentheses in the respective figure legend.

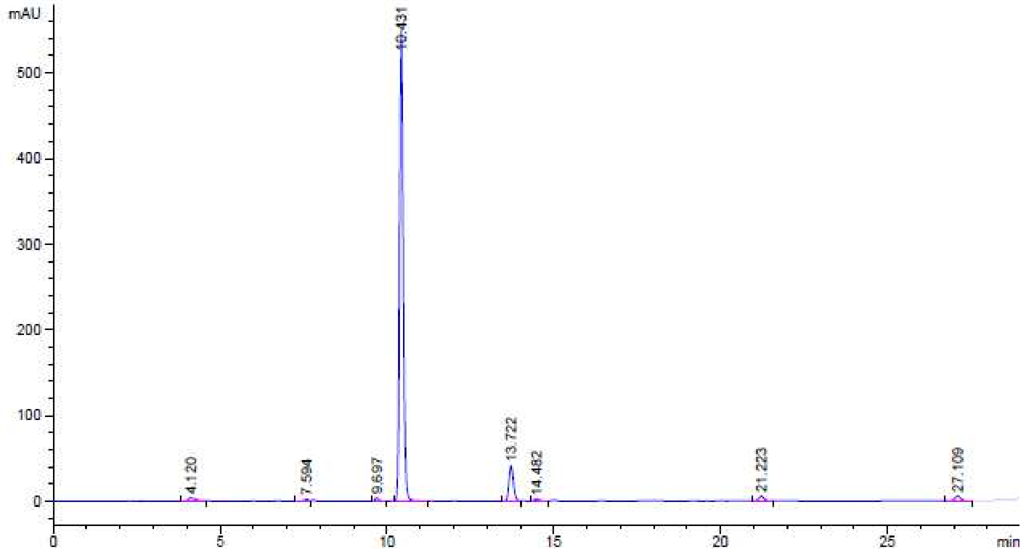

Figure S1. Chromatogram of extract 1 (P = 120 bar, cosolvent: water).

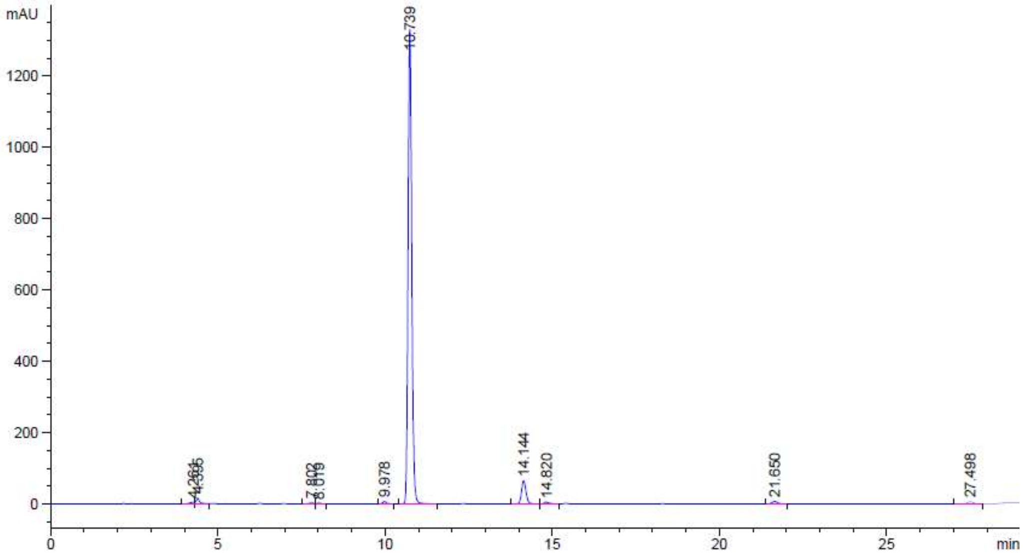

Figure S2. Chromatogram of extract 2 (P = 120 bar, cosolvent: ethanol).

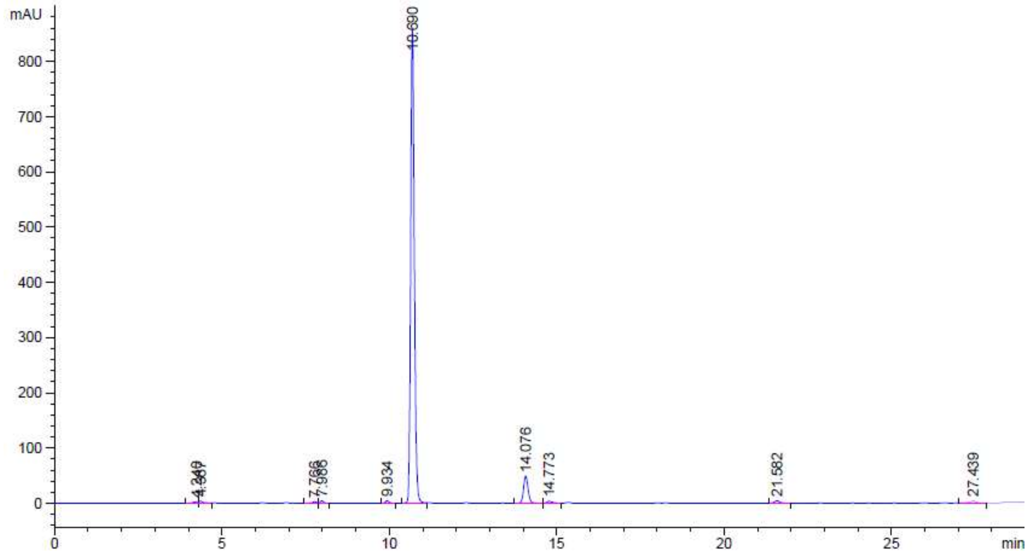

Figure S3. Chromatogram of extract 3 (P = 240 bar, cosolvent: water).

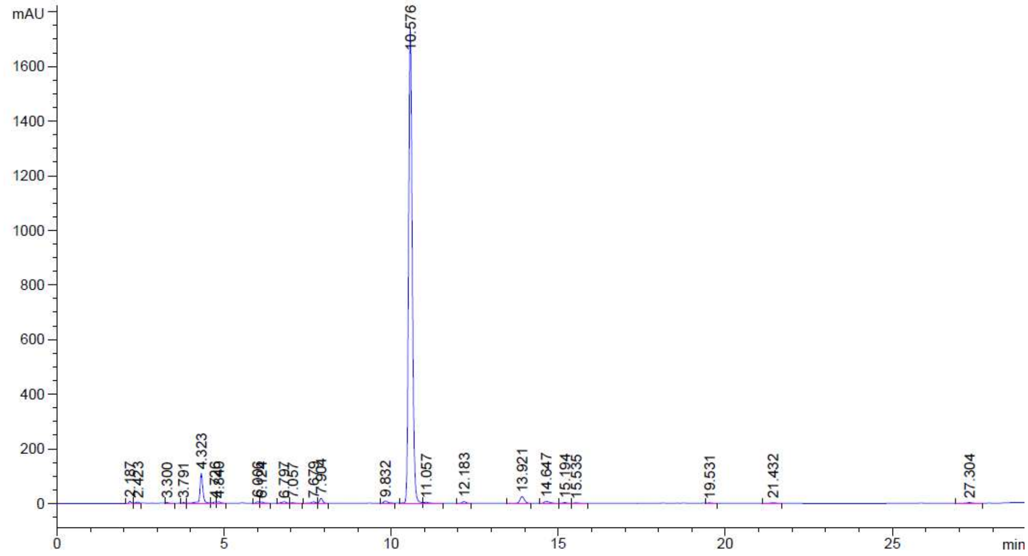

Figure S4. Chromatogram of extract 4 (P = 240 bar, cosolvent: ethanol).

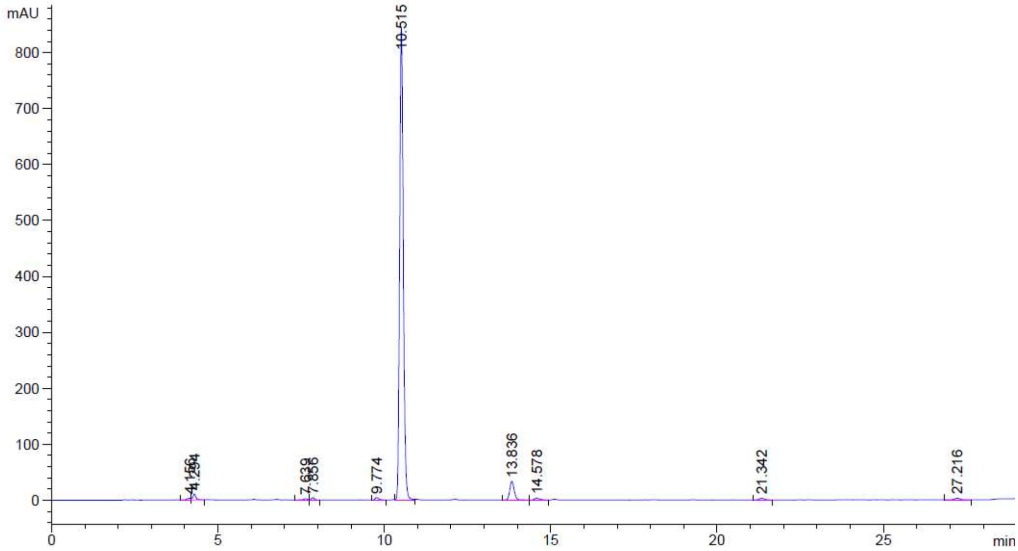

Figure S5. Chromatogram of extract 5 (P = 180 bar, cosolvent: ethanol:water).

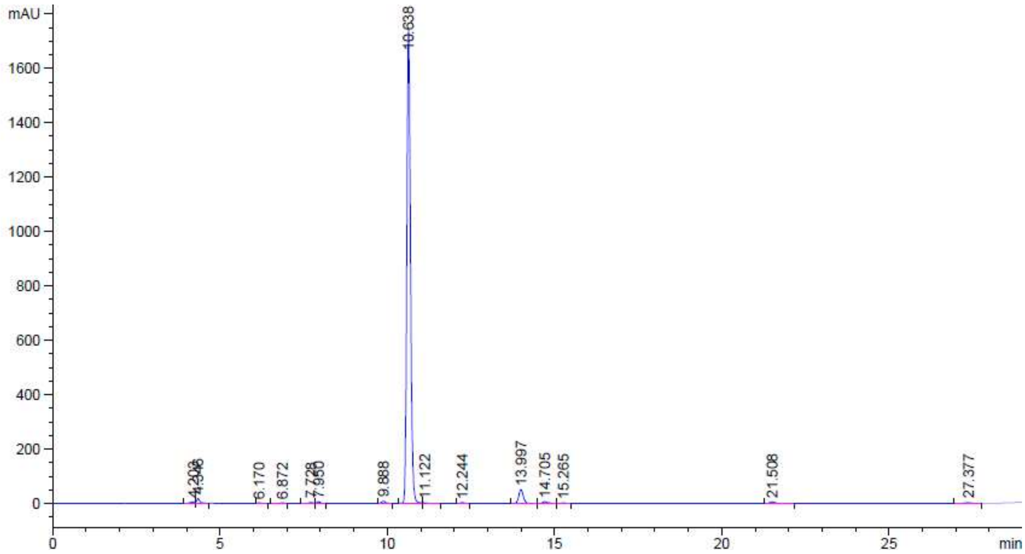

Figure S6. Chromatogram of extract 6 (P = 180 bar, cosolvent: ethanol:water).

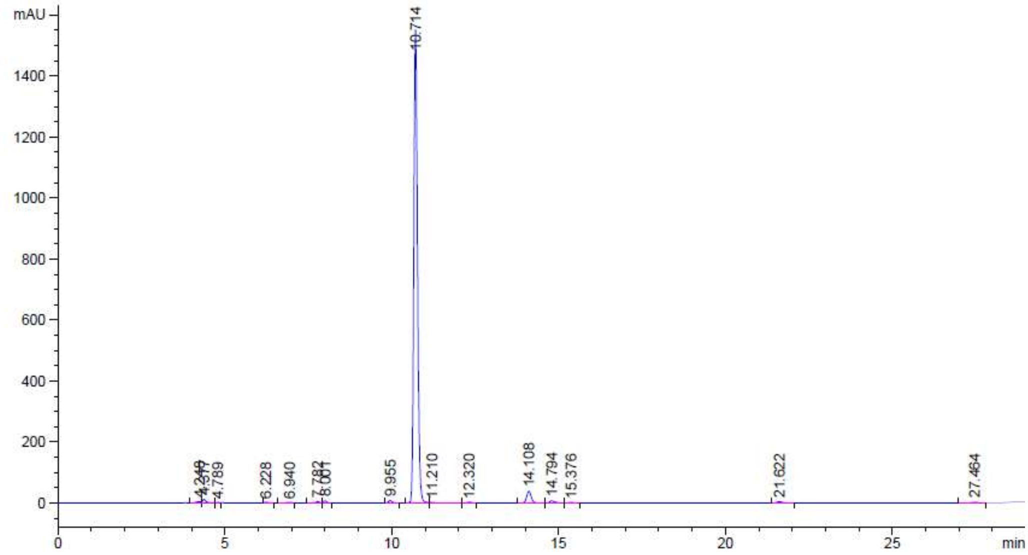

Figure S7. Chromatogram of extract 7 (P = 180 bar, cosolvent: ethanol:water).

Figures S8 and S9 present analysis results after extract purification. The compound *p*-anisic acid was concentrated at subfractions 4 and 5 that was evidenced by HPLC analysis (Figure S8).

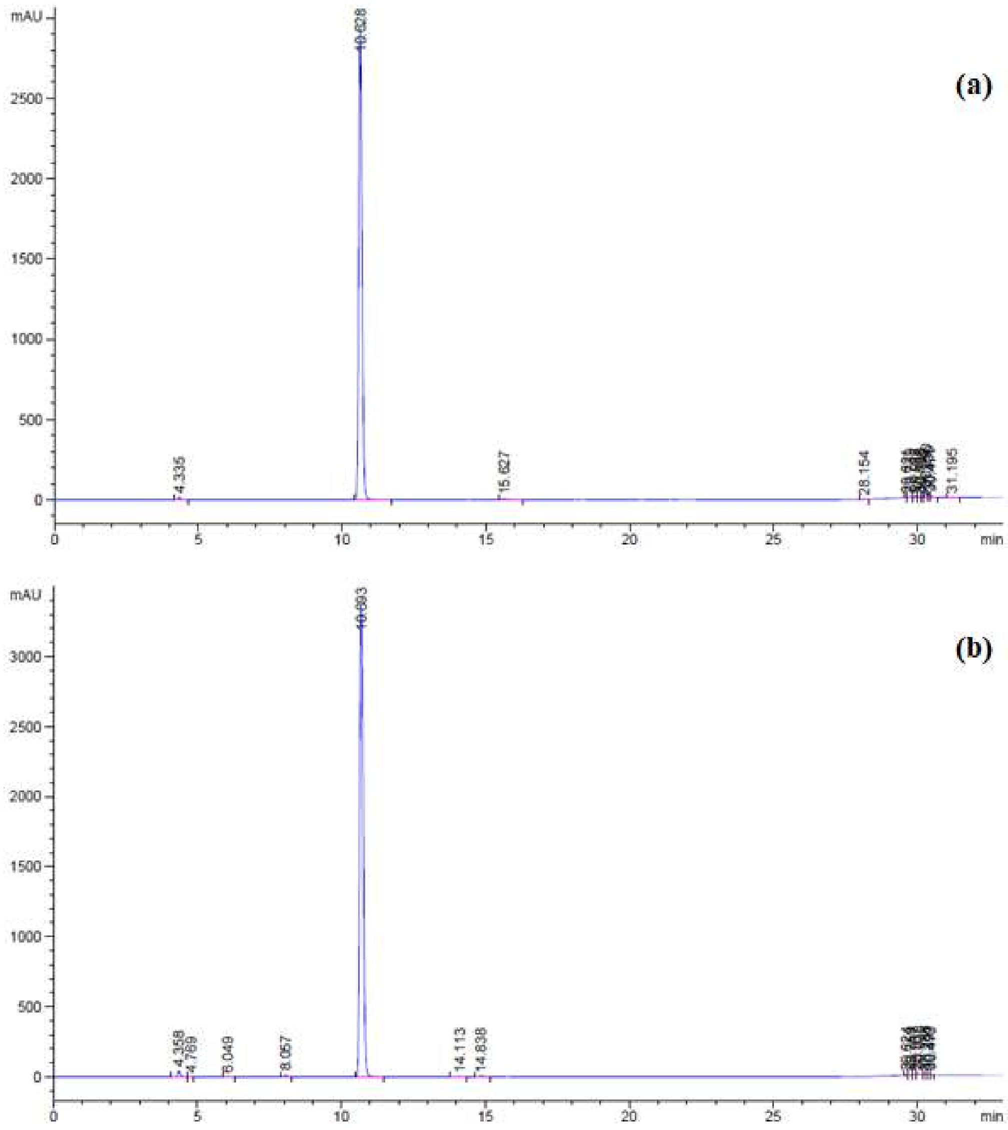

Figure S8. Chromatograms of (**a**) subfraction 4 and (**b**) subfraction 5 were obtained by silica gel column chromatography separation.

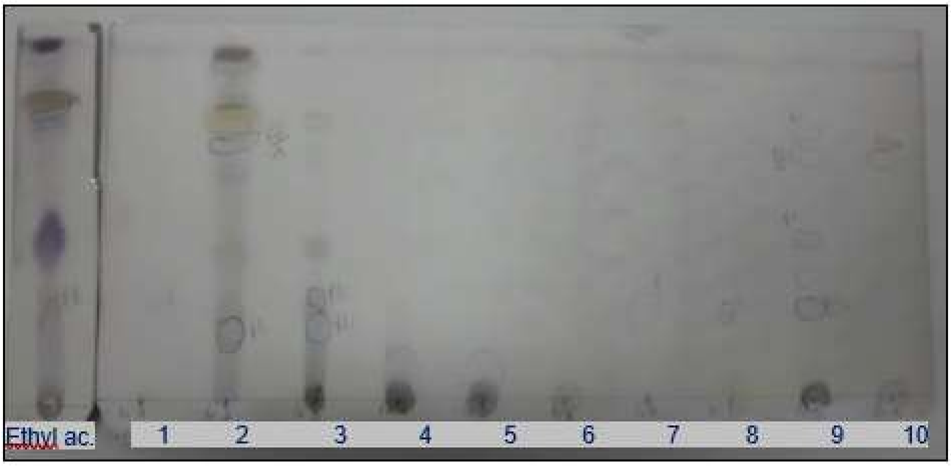

Figure S9. TLC for the ethyl acetate fraction and subfractions 1–10 after application of sulfuric vanillin as color reagent (the marks drawn represent the spots visualized under U.V. light).

Filename:	molecules-1532240-supplementary.docx
Directory:	C:\Users\MDPI\Documents
Template:	C:\Users\MDPI\AppData\Roaming\Microsoft\Templates\Normal.dotm
Title:	
Subject:	
Author:	Graciane Fabiela da Silva
Keywords:	
Comments:	
Creation Date:	1/25/2022 4:28:00 AM
Change Number:	2
Last Saved On:	1/25/2022 4:28:00 AM
Last Saved By:	Eduardo Cassel
Total Editing Time:	0 Minutes
Last Printed On:	1/30/2022 5:00:00 PM
As of Last Complete Printing	
Number of Pages:	5
Number of Words:	197 (approx.)
Number of Characters:	1,078 (approx.)
